# Comparison of linear and semi-parametric models incorporating genomic, pedigree, and associated loci information for the prediction of resistance to stripe rust in an Austrian winter wheat breeding program

**DOI:** 10.1007/s00122-023-04249-6

**Published:** 2023-01-24

**Authors:** Laura Morales, Christian Ametz, Hermann Gregor Dallinger, Franziska Löschenberger, Anton Neumayer, Simone Zimmerl, Hermann Buerstmayr

**Affiliations:** 1 Institute of Biotechnology in Plant Production, Department of Agrobiotechnology, University of Natural Resources and Life Sciences Vienna, Tulln, Austria; 2 Saatzucht Donau GmbH and CoKG, Probstdorf, Austria

## Abstract

**Key message:**

We used a historical dataset on stripe rust resistance across 11 years in an Austrian winter wheat breeding program to evaluate genomic and pedigree-based linear and semi-parametric prediction methods.

**Abstract:**

Stripe rust (yellow rust) is an economically important foliar disease of wheat (*Triticum aestivum* L.) caused by the fungus *Puccinia striiformis* f. sp. *tritici*. Resistance to stripe rust is controlled by both qualitative (R-genes) and quantitative (small- to medium-effect quantitative trait loci, QTL) mechanisms. Genomic and pedigree-based prediction methods can accelerate selection for quantitative traits such as stripe rust resistance. Here we tested linear and semi-parametric models incorporating genomic, pedigree, and QTL information for cross-validated, forward, and pairwise prediction of adult plant resistance to stripe rust across 11 years (2008–2018) in an Austrian winter wheat breeding program. Semi-parametric genomic modeling had the greatest predictive ability and genetic variance overall, but differences between models were small. Including QTL as covariates improved predictive ability in some years where highly significant QTL had been detected via genome-wide association analysis. Predictive ability was moderate within years (cross-validated) but poor in cross-year frameworks.

**Supplementary Information:**

The online version contains supplementary material available at 10.1007/s00122-023-04249-6.

## Introduction

The fungus *Puccinia striiformis* f. sp. *tritici* (*Pst*) causes stripe rust (yellow rust), an economically important foliar disease of wheat (*Triticum aestivum* L.). Resistance breeding is the most effective strategy for combating stripe rust epidemics (Chen 2020). Resistance to stripe rust in wheat is both qualitatively and quantitatively inherited (Rosewarne et al. 2008; Zegeye et al. 2014; Waqar et al. 2018; Blake et al. 2019; Ye et al. 2019). While most *Yr* genes confer complete (qualitative) resistance against specific *Pst* races and favorable *Yr* alleles can be efficiently deployed via marker assisted selection (MAS), *Yr*-mediated resistance can be easily overcome by rapidly evolving *Pst* populations (Poland et al. 2009; Buerstmayr et al. 2014; Hovmøller et al. 2016; Chen 2020; Klymiuk et al. 2020; Tehseen et al. 2020). Quantitative trait loci (QTL) and adult plant resistance (APR) *Yr* genes provide partial, race non-specific resistance that can be more durable in comparison with race-specific *Yr* genes (Poland et al. 2009; Chen 2020) but the effects of quantitative resistance mechanisms can be epistatically masked in the presence of race-specific *Yr* resistance alleles (Poland and Rutkoski 2016; Michel et al. 2022). Selection for and pyramiding of resistance QTL and APR genes via MAS can be an efficient strategy for achieving high levels of quantitative resistance (Ragimekula et al. 2013; Poland and Rutkoski 2016; Chen 2020).

Genomic prediction is a powerful tool for plant breeding, accelerating the breeding cycle and increasing genetic gain for quantitative traits (Heffner et al. 2010; Heslot et al. 2015; Poland and Rutkoski 2016; Crossa et al. 2017). Both linear and semi-parametric modeling have been shown to accurately predict stripe rust resistance (Juliana et al. 2017; Muleta et al. 2017; Tehseen et al. 2021; Shahinnia et al. 2022), but semi-parametric methods can improve prediction accuracy under epistatic interactions (Gianola et al. 2006; Gianola and Van Kaam 2008; Heslot et al. 2012; Juliana et al. 2017). Incorporating known QTL as model covariates can also enhance prediction accuracy for stripe rust resistance (Juliana et al. 2017; Shahinnia et al. 2022) and other quantitative disease resistance traits (Poland and Rutkoski 2016).

Prediction modeling for stripe rust has been previously assessed only under cross-validation and in highly controlled, artificially inoculated experiments with limited numbers of genotypes and environments (Juliana et al. 2017; Muleta et al. 2017; Tehseen et al. 2021; Shahinnia et al. 2022). Stripe rust resistance mechanisms in an active wheat breeding program can be influenced not only by genetic changes in the wheat population as a result of breeders’ decisions, but also by genetic changes in rapidly evolving *Pst* populations. As such, the evaluation of prediction models for stripe rust resistance should reflect these dynamic and interacting processes.

Here, we tested the predictive ability of linear and semi-parametric models incorporating genomic, pedigree, and QTL information on the prediction of stripe rust resistance under cross-validation and various cross-population frameworks using a historical dataset on more than 5000 Austrian winter wheat breeding lines evaluated over 11 years, largely under natural *Pst* infection (Morales et al. 2021). Linear models included genomic and pedigree-based best linear unbiased prediction (GBLUP and PBLUP, respectively) (Meuwissen et al. 2001; Endelman and Jannink 2012) and non-parametric models included genomic and pedigree-based reproducing kernel Hilbert spaces prediction (GRKHS and PRKHS, respectively) (Gianola et al. 2006; Gianola and Van Kaam 2008; González-Camacho et al. 2012). The QTL used as prediction model covariates in this study had been previously identified via genome-wide association (GWA) in this material (Morales et al. 2021).

## Materials and methods

### Phenotypic, genotypic, and pedigree data

Here we analyzed a historical stripe rust dataset from the winter wheat breeding program of Saatzucht Donau GmbH & CoKG (Probstdorf, Austria), as described previously by Morales et al. (2021). Briefly, 20,529 genotypes were evaluated for adult stripe rust resistance on a 1 (most resistant) to 9 (most susceptible) scale in 71 trials across 53 locations from 2008 to 2018, where the majority (60/71 trials) of the trials were naturally infected by *Pst* (Morales et al. 2021). The phenotypic dataset is highly unbalanced, with most genotypes only evaluated in one plot in one trial (Morales et al. 2021). Within-trial spatial variation in stripe rust severity was adjusted using the “SpATS” package (Rodríguez-Álvarez et al. 2018) in R Core Team (2020), Morales et al. (2021). Within and across years, a mixed model was fit with the spatially-adjusted stripe rust plot values as the response, genotype as a fixed effect, and trial as a random effect using the “breedR” package (Muñoz and Sanchez 2020) in R Core Team (2020) and the genotype best linear unbiased estimates (BLUEs) were then extracted from the model for further analysis (Morales et al. 2021). The within- and across-year genotype BLUEs (Morales et al. 2021) were used for genomic and pedigree-based prediction in this study (Online Resource 1). For prediction models including multiple years in the training set, we fit mixed models with the spatially-adjusted stripe rust plot values from the years in the training set as the response, genotype as a fixed effect, and trial as a random effect using the “breedR” package (Muñoz and Sanchez 2020) in R Core Team (2020) and then extracted the BLUEs for further analysis (Online Resource 1).

Pedigree information was available for 41,461 individuals (Online Resource 2). A subset of 5233 lines selected based on good agronomic performance, grain quality, and disease resistance had also been genotyped with 9744 single nucleotide polymorphisms (SNPs) derived from a custom 6 K Illumina marker array (Illumina, Inc., San Diego, CA, USA) and DArTseq (Diversity Arrays Technology Pty Ltd, Canberra, Australia) genotyping-by-sequencing (Akbari et al. 2006; Elshire et al. 2011) technology (Morales et al. 2021) (Online Resource 3). SNP genotypes were coded in terms of alternate alleles “a” and “A,” where − 1 = aa (homozygous “a” allele), 0 = Aa (heterozygous), and 1 = AA (homozygous “A” allele), and missing SNP data were imputed with the “missForest” package (Stekhoven and Bühlmann 2012) in R (Morales et al. 2021; R Core Team 2020) (Online Resource 3).

Morales et al. (2021) previously identified 150 SNPs that were significantly associated with stripe rust resistance within 2009, 2010, 2011, 2014, 2015, 2018, and across 2008–2018 in this dataset, representing 56 QTL (Online Resource 4). For years in which no SNPs were significantly detected (2008, 2012, 2013, 2016, 2017), we selected the SNPs with the lowest p-values (Online Resource 4).

### Prediction models

All statistical analyses were conducted in R Core Team (2020). For genomic and pedigree-based best linear unbiased prediction (GBLUP and PBLUP, respectively) and genomic and pedigree-based reproducing kernel Hilbert spaces prediction (GRKHS and PRKHS, respectively), we used the “breedR” package (Muñoz and Sanchez 2020) to fit the following mixed model (Meuwissen et al. 2001):

$${\varvec{y}}={1}_{n}{\varvec{\mu}}+{\varvec{Z}}{\varvec{u}}+{\varvec{\varepsilon}},$$

where ***y*** is the vector of genotype BLUEs for stripe rust resistance, ***µ*** is the vector of overall means, ***Z*** is the design matrix of random effects, ***u*** is the vector of genotype random effects (${\varvec{u}}\sim N(0, {\varvec{K}}{\sigma }_{a}^{2}$)), and ***ε*** is the vector of residuals (${\varvec{\varepsilon}}\sim N(0,{\varvec{I}}{\sigma }_{\varepsilon }^{2}$)). The variance of the genotype term was modeled as ***K****σ*^*2*^_*a*_, where ***K*** is the realized additive relationship matrix (Endelman and Jannink 2012) and *σ*^*2*^_*a*_ is the estimated additive genetic variance (Yu et al. 2006). For each GBLUP model, we calculated ***K*** using SNP data from the lines included in the model with the “rrBLUP” package (Endelman and Jannink 2012). We used pedigree data to estimate ***K*** for all lines using the “AGHmatrix” package (Amadeu et al. 2016) and ***K*** was then subset for the lines included in each PBLUP model and which had also been genotyped. Using SNP data from the lines included in each GRKHS model and the pedigree ***K*** matrix subset for the lines in each PRKHS model and which had also been genotyped, we used the “BGGE” package (Granato et al. 2018) to model ***K*** as the following reproducing Gaussian kernel:

$$\mathrm{K}\left({x}_{i},{x}_{j}\right)=\mathrm{exp}\left(\frac{-{\sum }_{k}{\left({x}_{ik}-{x}_{jk}\right)}^{2}}{q}\right),$$

where the numerator is the Euclidian distance between individuals based on SNPs (GRKHS) (González-Camacho et al. 2012) or twice the coefficient of ancestry (PRKHS) (Juliana et al. 2017), scaled by the percentile of the square of the Euclidean distance *q* (González-Camacho et al. 2012).

In addition, we incorporated the QTL previously identified via GWA (Morales et al. 2021) in each prediction model (GBLUP-A, PBLUP-A, GRKHS-A, PRKHS-A). For the across-year models and each within-year model, QTL significantly associated with stripe rust across years or in that year (Morales et al. 2021), respectively, were included as fixed covariates. Similarly, for pairwise and forward prediction, the QTL associated in the test year(s) were included as covariates. The following mixed model (Meuwissen et al. 2001) was fit with the “breedR” package (Muñoz and Sanchez 2020):

$${\varvec{y}}={1}_{n}{\varvec{\mu}}+{\varvec{X}}{\beta }_{i}\dots {\varvec{X}}{\beta }_{j}+{\varvec{Z}}{\varvec{u}}+{\varvec{\varepsilon}},$$

where ***y*** is the vector of genotype BLUEs for stripe rust resistance, ***µ*** is the vector of overall means, ***X***_***i…j***_ are the matrices of SNPs *i* to *j*, *β*_*i*…j_ are the fixed effects of SNPs *i* to *j*, ***Z*** is the design matrix of random effects, ***u*** is the vector of genotype random effects (${\varvec{u}}\sim N(0, {\varvec{K}}{\sigma }_{a}^{2}$)), and ***ε*** is the vector of residuals (${\varvec{\varepsilon}}\sim N(0,{\varvec{I}}{\sigma }_{\varepsilon }^{2}$)). Covariance structures were specified as described previously.

We also conducted ordinary least squares (OLS) regression using a similar approach as described above, with the only difference being that the random genotypic term was not included. The following mixed model (Meuwissen et al. 2001) was fit with the “breedR” package (Muñoz and Sanchez 2020):

$${\varvec{y}}={1}_{n}{\varvec{\mu}}+{\varvec{X}}{\beta }_{i}\dots {\varvec{X}}{\beta }_{j}+{\varvec{\varepsilon}},$$

where ***y*** is the vector of genotype BLUEs for stripe rust resistance, ***µ*** is the vector of overall means, ***X***_***i…j***_ are the matrices of SNPs *i* to *j*, *β*_*i*…j_ are the fixed effects of SNPs *i* to *j*, and ***ε*** is the vector of residuals (${\varvec{\varepsilon}}\sim N(0,{\varvec{I}}{\sigma }_{\varepsilon }^{2}$)).

### Prediction frameworks

We used cross-validation (five-fold, 10 replications) to evaluate each prediction model within and across years. Within each fold of each replication of each model, the response vector ***y*** included the genotype BLUEs of the training set and missing values for the test set. ***K*** was estimated using SNP data from all genotypes in both the training and test sets. Predictive ability was defined as the Pearson’s correlation between the observed and predicted values of the test set in each fold of each replication. For each model within and across years, we estimated heritability within each replication/fold as the proportion of the total variance explained by the random genotypic term.

Because GRKHS had the best predictive ability and highest heritability overall in the cross-validated analysis and because GBLUP is the most commonly used model for genomic prediction (Zhang et al. 2021), we conducted further cross-year testing on GRKHS and GBLUP. In the forward prediction framework, BLUES within each year (2009–2010) were used as the test set. For each test set, we comprised progressive training set(s) of BLUEs from the previous year(s), with the first training set only including the year immediately before the test year and the last training set including all years prior to the test year. For example, the training sets for the 2011 test year included BLUEs from 2010, 2009–2010, and 2008–2010. We also evaluated GBLUP and GRKHS between pairs of years. For each pair of years, one year was used as the training set and the other year as the test set, and vice versa. The forward prediction and between-year test sets were selected in two ways: (1) all genotypes in the training and test sets were included (“overlap”) and (2) genotypes that were present in both the training and test sets were excluded from the test set (“no overlap”). For each model, the response vector ***y*** included the genotype BLUEs of the training set and missing values for the test set. ***K*** was estimated using SNP data from all genotypes in both the training and test sets. Predictive ability was defined as the Pearson’s correlation between the observed and predicted values of the test set and heritability was estimated as the proportion of the total variance explained by the random genotypic term.

## Results

### Comparison of cross-validated stripe rust prediction models within and across years

Cross-validated predictive ability (* PA*) for stripe rust was moderate, with a grand mean of * PA* = 0.40 ± 0.24. The number of lines per year ranged from 47 to 1639 (Table 2). Overall, the difference in predictive ability among kinship-based models (all models except OLS) was small, ranging from *PA* = 0.37 for PBLUP to *PA =* 0.49 for GRKHS-A, while OLS had the lowest predictive ability (*PA* = 0.22) (Table 1). GRKHS and GRKHS-A had the greatest heritability (*h*^2^ = 0.75–0.79), while GBLUP and GBLUP-A had the lowest heritability (*h*^2^ = 0.34–0.42) (Table 2). In an overall comparison among years, predictive ability was highest across years and within 2014 and 2016 (* PA*= 0.57–0.58) and lowest within 2008 and 2009 (*PA *= 0.29–0.30), while heritability was highest within 2009 (*h*^2^ = 0.84) and lowest within 2011 (*h*^2^ = 0.26) (Tables 1 and 2). Predictive ability and heritability were weakly positively correlated (*r* = 0.09, *p* = 9 × 10^–11^).

**Table 1:** Predictive ability of cross-validated stripe rust prediction models within and across years (2008–2018)

Year	OLS	GBLUP	GBLUP-A	GRKHS	GRKHS-A	PBLUP	PBLUP-A	PRKHS	PRKHS-A	Overall
2008	0.35 ± 0.42 ab	0.23 ± 0.32 bc	0.39 ± 0.34 ab	0.34 ± 0.24 ab	0.50 ± 0.27 a	0.03 ± 0.27 c	0.37 ± 0.40 ab	0.06 ± 0.25 c	0.35 ± 0.42 ab	*0.29* ± *0.36 f*
2009	−0.12 ± 0.17 e	0.46 ± 0.14 a	0.41 ± 0.20 ab	0.41 ± 0.15 abc	0.42 ± 0.17 ab	0.30 ± 0.16 cd	0.22 ± 0.16 d	0.32 ± 0.16 bcd	0.25 ± 0.18 d	*0.30* ± *0.23 df*
2010	0.19 ± 0.18 c	0.45 ± 0.13 a	0.38 ± 0.08 ab	0.43 ± 0.12 ab	0.37 ± 0.14 b	0.40 ± 0.10 ab	0.38 ± 0.13 ab	0.41 ± 0.11 ab	0.38 ± 0.15 ab	*0.38* ± *0.15 c*
2011	0.13 ± 0.23 b	0.25 ± 0.13 a	0.21 ± 0.20 ab	0.27 ± 0.15 a	0.29 ± 0.20 a	0.24 ± 0.14 ab	0.20 ± 0.20 ab	0.22 ± 0.15 ab	0.22 ± 0.24 ab	*0.23* ± *0.19 e*
2012	0.33 ± 0.11 c	0.44 ± 0.11 b	0.47 ± 0.10 ab	0.48 ± 0.10 ab	0.51 ± 0.10 a	0.45 ± 0.10 ab	0.49 ± 0.10 ab	0.43 ± 0.09 b	0.48 ± 0.08 ab	*0.45* ± *0.11 b*
2013	0.17 ± 0.11 b	0.39 ± 0.12 a	0.42 ± 0.12 a	0.42 ± 0.13 a	0.44 ± 0.10 a	0.42 ± 0.13 a	0.46 ± 0.12 a	0.41 ± 0.12 a	0.43 ± 0.11 a	*0.40* ± *0.14 c*
2014	0.56 ± 0.04 d	0.63 ± 0.03 c	0.64 ± 0.03 bc	0.65 ± 0.04 ab	0.68 ± 0.04 a	0.42 ± 0.04 e	0.63 ± 0.04 bc	0.42 ± 0.05 e	0.63 ± 0.04 bc	*0.58* ± *0.10 a*
2015	0.36 ± 0.08 d	0.41 ± 0.06 c	0.45 ± 0.07 ab	0.44 ± 0.06 abc	0.48 ± 0.07 a	0.27 ± 0.06 e	0.41 ± 0.07 bc	0.29 ± 0.06 e	0.41 ± 0.08 c	*0.39* ± *0.10 c*
2016	0.15 ± 0.05 e	0.59 ± 0.04 cd	0.59 ± 0.03 d	0.63 ± 0.03 ab	0.64 ± 0.02 a	0.61 ± 0.03 c	0.61 ± 0.03 bc	0.65 ± 0.04 a	0.65 ± 0.03 a	*0.57* ± *0.16 a*
2017	−0.05 ± 0.14 d	0.41 ± 0.08 ab	0.40 ± 0.09 abc	0.41 ± 0.10 ab	0.41 ± 0.09 a	0.37 ± 0.08 abc	0.36 ± 0.08 abc	0.35 ± 0.08 bc	0.35 ± 0.07 c	*0.33* ± *0.17 d*
2018	0.14 ± 0.11 e	0.39 ± 0.05 d	0.40 ± 0.05 d	0.44 ± 0.06 c	0.45 ± 0.04 bc	0.45 ± 0.06 bc	0.46 ± 0.06 abc	0.48 ± 0.05 ab	0.49 ± 0.04 a	*0.41* ± *0.12 c*
Across	0.44 ± 0.02 e	0.59 ± 0.02 c	0.60 ± 0.02 bc	0.63 ± 0.02 a	0.64 ± 0.01 a	0.54 ± 0.02 d	0.61 ± 0.02 b	0.59 ± 0.02 c	0.63 ± 0.02 a	*0.58* ± *0.06 a*
*Overall*	*0.22* ± *0.25 d*	*0.44* ± *0.17 b*	*0.45* ± *0.18 b*	*0.46* ± *0.16 ab*	*0.49* ± *0.17 a*	*0.38* ± *0.19 c*	*0.44* ± *0.20 b*	*0.39* ± *0.19 c*	*0.44* ± *0.21 b*	

Each model was five-fold cross-validated with 10 replications within years and across years. Predictive ability refers to the Pearson’s correlation between the observed and predicted values of the test set within each fold of each replication. Means ± standard deviations of predictive ability are displayed. For comparisons among models within each year and overall, groups within each table row that are not connected by the same letter have significantly different predictive ability (Tukey’s HSD test,* p* < 0.05). For overall comparisons among years, groups within the last table column that are not connected by the same letter have significantly different predictive ability (Tukey’s HSD test,* p* < 0.05). *OLS* ordinary least squares.* GBLUP* genomic best linear unbiased prediction.* GBLUP-A* GBLUP with QTL covariates.* GRKHS* genomic reproducing kernel Hilbert spaces prediction.* GRKHS-A* GRKHS with QTL covariates.* PBLUP* pedigree-based best linear unbiased prediction.* PBLUP-A* PBLUP with QTL covariates.* PRKHS* pedigree-based reproducing kernel Hilbert spaces prediction.* PRKHS-A* PRKHS with QTL covariates

*p* < 0.05 are italicized

**Table 2:** Heritability of cross-validated stripe rust prediction models within and across years (2008–2018)

Year	N	GBLUP	GBLUP-A	GRKHS	GRKHS-A	PBLUP	PBLUP-A	PRKHS	PRKHS-A	Overall
2008	47	0.70 ± 0.34 b	0.26 ± 0.39 c	0.98 ± 0.07 a	0.77 ± 0.35 b	0.31 ± 0.35 c	0.18 ± 0.34 c	0.15 ± 0.33 c	0.11 ± 0.31 c	*0.44* ± *0.45 f*
2009	93	0.54 ± 0.19 d	0.78 ± 0.15 c	0.86 ± 0.12 b	0.99 ± 0.04 a	0.89 ± 0.10 b	0.92 ± 0.08 ab	0.86 ± 0.14 b	0.90 ± 0.12 b	*0.84* ± *0.18 a*
2010	182	0.33 ± 0.09 e	0.35 ± 0.08 e	0.63 ± 0.14 cd	0.68 ± 0.11 bc	0.76 ± 0.11 a	0.68 ± 0.15 bc	0.75 ± 0.14 ab	0.59 ± 0.16 d	*0.60* ± *0.20 de*
2011	243	0.26 ± 0.12 cd	0.08 ± 0.05 f	0.51 ± 0.13 a	0.31 ± 0.07 bc	0.32 ± 0.13 b	0.25 ± 0.08 d	0.12 ± 0.05 ef	0.16 ± 0.03 e	*0.26* ± *0.16 h*
2012	288	0.45 ± 0.08 e	0.39 ± 0.07 f	0.86 ± 0.07 a	0.83 ± 0.07 ab	0.79 ± 0.06 b	0.68 ± 0.10 c	0.65 ± 0.07 c	0.60 ± 0.08 d	*0.66* ± *0.18 bc*
2013	266	0.43 ± 0.08 d	0.39 ± 0.07 d	0.87 ± 0.06 a	0.85 ± 0.06 ab	0.82 ± 0.11 b	0.88 ± 0.08 a	0.58 ± 0.09 c	0.55 ± 0.08 c	*0.67* ± *0.21 bc*
2014	1458	0.58 ± 0.03 e	0.31 ± 0.03 g	0.97 ± 0.01 a	0.89 ± 0.04 b	0.49 ± 0.05 f	0.47 ± 0.06 f	0.67 ± 0.04 c	0.62 ± 0.06 d	*0.63* ± *0.21 cd*
2015	1298	0.51 ± 0.04 e	0.31 ± 0.06 f	0.97 ± 0.01 a	0.91 ± 0.03 a	0.68 ± 0.04 c	0.61 ± 0.07 d	0.81 ± 0.14 b	0.34 ± 0.19 f	*0.64* ± *0.25 cd*
2016	1387	0.43 ± 0.02 d	0.44 ± 0.03 d	0.83 ± 0.02 b	0.83 ± 0.02 b	0.65 ± 0.02 c	0.64 ± 0.02 c	0.86 ± 0.01 a	0.86 ± 0.01 a	*0.69* ± *0.17 b*
2017	638	0.32 ± 0.06 c	0.33 ± 0.08 c	0.69 ± 0.09 a	0.69 ± 0.10 a	0.69 ± 0.05 a	0.69 ± 0.05 a	0.57 ± 0.15 b	0.56 ± 0.12 b	*0.57* ± *0.18 e*
2018	1639	0.21 ± 0.04 d	0.20 ± 0.01 d	0.54 ± 0.04 a	0.51 ± 0.03 b	0.29 ± 0.02 c	0.29 ± 0.02 c	0.54 ± 0.03 a	0.53 ± 0.03 a	*0.39* ± *0.15 g*
Across	5233	0.34 ± 0.01 f	0.27 ± 0.01 g	0.79 ± 0.02 b	0.73 ± 0.02 c	0.64 ± 0.02 d	0.58 ± 0.02 e	0.86 ± 0.01 a	0.79 ± 0.02 b	*0.63* ± *0.21 cd*
*Overall*		*0.42* ± *0.19 e*	*0.34* ± *0.21 f*	*0.79* ± *0.17 a*	*0.75* ± *0.21 a*	*0.61* ± *0.24 bc*	*0.57* ± *0.26 cd*	*0.62* ± *0.27 b*	*0.55* ± *0.27 d*	

Each model was five-fold cross-validated with 10 replications within years and across years. Heritability was estimated as the proportion of the total variance explained by the random genotypic term within each fold of each replication. Means ± standard deviations for heritability are displayed. For comparisons among models within each year and overall, groups within each table row that are not connected by the same letter have significantly different heritability (Tukey’s HSD test,* p* < 0.05). For overall comparisons among years, groups within the last table column that are not connected by the same letter have significantly different heritability (Tukey’s HSD test,* p* < 0.05).* OLS* ordinary least squares.* GBLUP* genomic best linear unbiased prediction.* GBLUP-A* GBLUP with QTL covariates.* GRKHS* genomic reproducing kernel Hilbert spaces prediction.* GRKHS-A* GRKHS with QTL covariates.* PBLUP* pedigree-based best linear unbiased prediction.* PBLUP-A* PBLUP with QTL covariates.* PRKHS* pedigree-based reproducing kernel Hilbert spaces prediction.* PRKHS-A* PRKHS with QTL covariates

*p* < 0.05 are italicized

Predictive ability was highest within 2009 and 2010 with GBLUP (Table 1). GRKHS best predicted 2011, while GRKHS-A had the best predictive ability within 2008, 2012, 2014, 2015, and 2017 (Table 1). Within 2016, GRKHS-A, PRKHS, and PRKHS-A has the highest predictive ability, while PRKHS-A best predicted 2018. In the across-years analysis, predictive ability was best with GRKHS, GRKHS-A, and PRKHS-A (Table 1). All kinship-based models performed equally within 2013 (Table 1).

For the genomic prediction methods, including QTL as covariates did not significantly improve predictive ability. Overall and within/across years, GBLUP and GBLUP-A performed equally, as did GRKHS and GRKHS-A (Table 1). However, pedigree-based models that included QTL covariates had higher predictive ability than their counterparts without QTL covariates in some cases. Overall, PBLUP-A and PRKHS-A had better predictive ability than PBLUP and PRKHS (Table 1). Similarly, PBLUP-A and PRKHS-A had higher predictive ability than PBLUP and PRKHS within 2008, 2014, and 2015, and across years (Table 1). Within 2008, 2014, and 2015, OLS had predictive ability comparable to or higher than PBLUP-A and PRKHS-A (Table 1).

### Comparison of between-year and forward prediction models for stripe rust

**Fig. 1:**
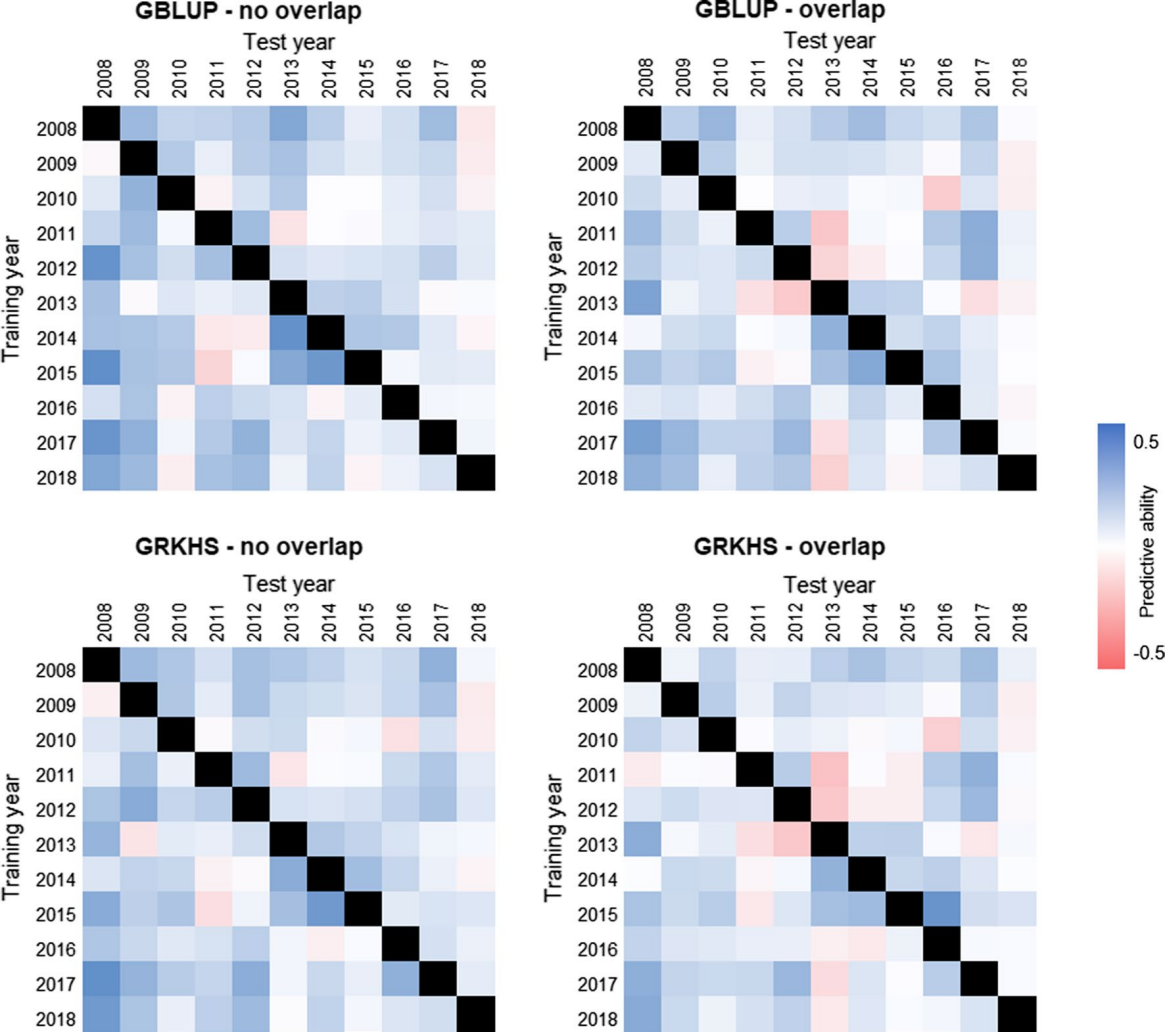
Between-year predictive ability of GBLUP and GRKHS models in which lines present in both the training and test sets were excluded from the test set (GBLUP–no overlap and GRKHS–no overlap) and in which all training and test set lines were included (GBLUP–overlap and GRKHS–overlap)

**Fig. 2:**
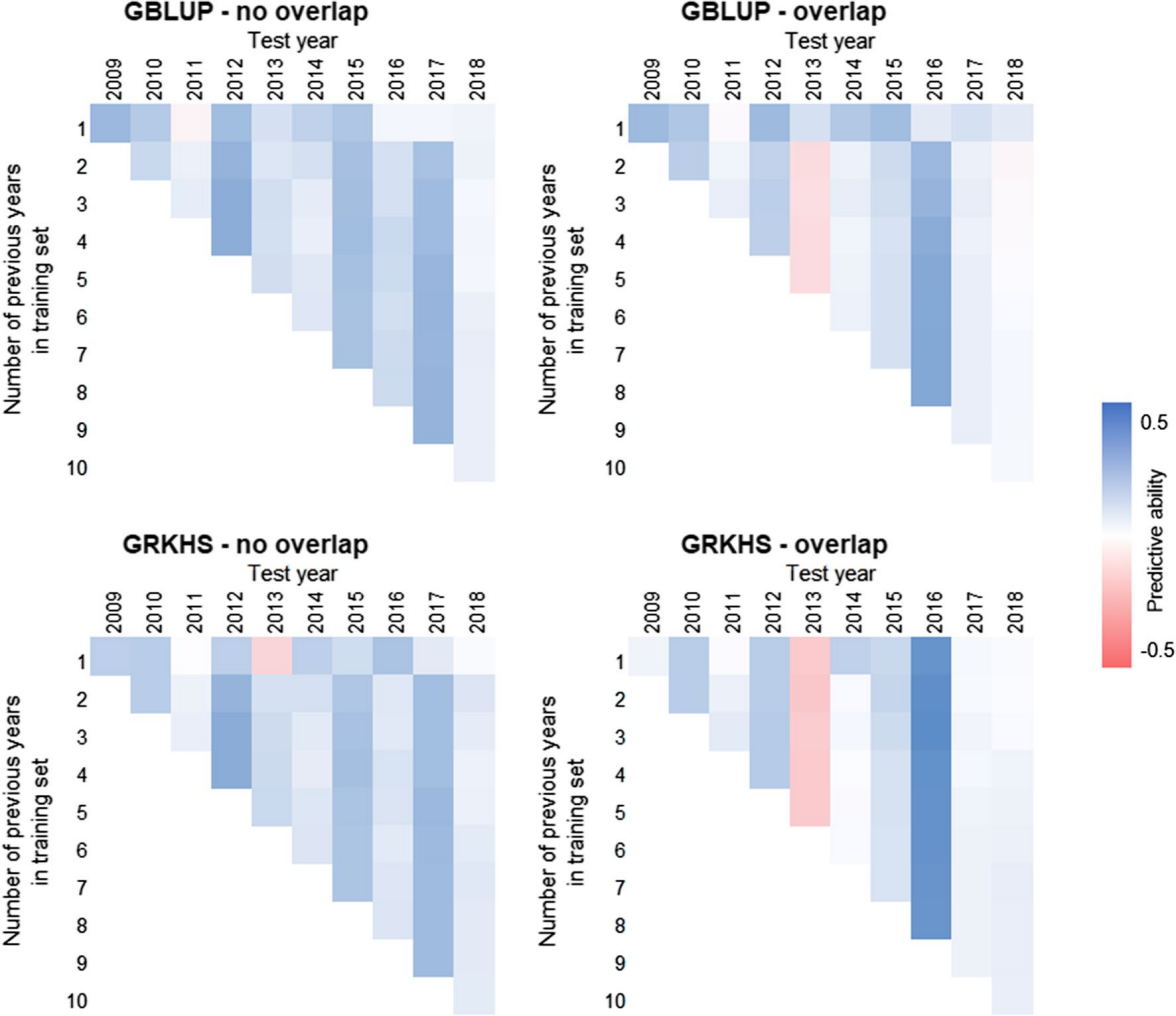
Forward predictive ability of GBLUP and GRKHS models in which lines present in both the training and test sets were excluded from the test set (GBLUP–no overlap and GRKHS–no overlap) and in which all training and test set lines were included (GBLUP–overlap and GRKHS–overlap). For each test year, the training sets comprised progressive sets of subsequent years

Both between-year and forward predictive ability were generally poor, with a grand mean of * PA* = 0.12 ± 0.14 for the between-year framework and * PA* = 0.14 ± 0.14 for forward prediction (Table 3, Figs. 1 and 2). Overall, the models in which genotypes present in both the training and test sets where excluded from the test set (GBLUP–no overlap; GRKHS–no overlap; * PA*_between_ = 0.14; * PA*_forward_ = 0.17) had better predictive ability than the models where all genotypes in the training and test sets were included (GBLUP–overlap; GRKHS–overlap; * PA*_between_ = 0.09–0.11; * PA*_forward_ = 0.10–0.12) (Table 3). Heritability was also higher with the “no overlap” models compared to the “overlap” models and the GBLUP models had greater heritability than their respective GRKHS models (Table 3).

**Table 3:** Predictive ability and heritability of between-year and forward prediction models for stripe rust

Framework	Model	Predictive ability	Heritability
Between-year	GBLUP – no overlap	0.141 ± 0.135 a	0.456 ± 0.166 b
	GBLUP – overlap	0.110 ± 0.128 ab	0.084 ± 0.158 d
	GRKHS – no overlap	0.143 ± 0.122 a	0.789 ± 0.172 a
	GRKHS – overlap	0.093 ± 0.125 b	0.229 ± 0.367 c
Forward	GBLUP – no overlap	0.170 ± 0.106 ab	0.397 ± 0.121 b
	GBLUP – overlap	0.099 ± 0.133 b	0.027 ± 0.065 c
	GRKHS – no overlap	0.167 ± 0.098 ab	0.802 ± 0.115 a
	GRKHS – overlap	0.115 ± 0.179 ab	0.101 ± 0.238 c

Predictive ability was calculated as the Pearson’s correlation between the observed and predicted values of the test set. Heritability was estimated as the proportion of the total variance explained by the random genotypic term. Means ± standard errors of predictive ability and heritability for each model as displayed. Groups that are not connected by the same letter have significantly different predictive ability or heritability (Tukey’s HSD test, *p* < 0.05). GBLUP – no overlap: genomic best linear unbiased prediction, in which lines present in both the training and test sets were excluded from the test set. GBLUP – overlap: GBLUP with all training and test set lines included. GRKHS – no overlap: genomic reproducing kernel Hilbert spaces prediction, in which lines present in both the training and test sets were excluded from the test set. GRKHS – overlap: GRKHS with all training and test set lines included

In the between-year framework, there was no consistent relationship between predictive ability and the number of years between the training and test sets (Pearson’s correlation *r* = 0.002; *p* = 0.9), although some trends were apparent. For example, the years 2013–2015 better predicted each other than other years (Fig. 1). Conversely, the training years 2008–2012 tended to have better predictive ability for the test years 2016–2017 than with other years, and vice versa (Fig. 1). The test year 2018 was poorly predicted by all training years (Fig. 1). The phenotypic correlation between pairs of years was generally higher than the corresponding genomic predictive ability (Fig. 1, Table 4). Adjacent pairs of years tended to have higher phenotypic correlations than pairs further apart in time (Table 4). The number of lines shared between pairs of years ranged from 14 to 541 (Table 4).

**Table 4:** Phenotypic correlations and number of shared genotypes between pairs of years from 2009–2018

n\r	2009	2010	2011	2012	2013	2014	2015	2016	2017	2018
2009	93	0.74***	0.53**	0.69**	0.50*	0.34	0.47*	0.56*	0.48	0.51
2010	68	182	0.24*	0.57**	0.51**	0.37*	0.32	0.30	0.02	0.36
2011	44	78	243	0.55***	0.27*	0.03	0.02	0.16	−0.18	0.09
2012	25	38	91	288	0.49***	0.100	0.28*	0.17	0.38*	0.25
2013	23	30	64	105	266	0.21*	0.55***	0.18	0.45**	0.17
2014	28	31	48	66	102	1458	0.49***	0.11	0.16	−0.01
2015	26	32	48	60	71	497	1298	0.02	0.30**	0.29**
2016	19	27	38	46	49	131	541	1380	0.24***	0.36***
2017	16	23	31	40	42	69	163	518	638	0.47***
2018	14	19	30	35	39	54	94	158	172	1639

Correlation coefficients (* r*) and * p*-values are in the upper diagonal. Numbers of genotypes (* n*) present in each pair of years are in the lower diagonal. Numbers of genotypes within each year are on the diagonal.* P*-values are denoted as *0.05 < * p*≤ 0.01; **0.01 < * p* 0.0001; * p* < 0.0001

In the forward prediction framework, we found no apparent trend with respect to the number of previous years in the training set versus predictive ability (Pearson’s correlation *r* = 0.1; *p* = 0.8). However, we did find trends in predictive ability among test years and models. With the GBLUP–no overlap and GRKHS–no overlap models, predictive ability was higher for the test years 2012 (*PA* = 0.34 ± 0.02), 2015 (*PA* = 0.26 ± 0.01), and 2017 (*PA*  = 0.29 ± 0.02) than other test years (*PA* = 0.06–0.19) (Fig. 2). While predictive ability for the test year 2016 was poor with the “no overlap” models (*PA* = 0.12 ± 0.03), its predictive ability with the “overlap” methods was moderate (*PA* = 0.41 ± 0.07) and comparable to cross-validated predictions (Fig. 2, Table 1). In contrast, prediction for the test year 2013 was very poor with the “overlap” models (*PA* = -0.14 ± 0.03) compared to the “no overlap” models (*PA* = 0.13 ± 0.02) (Fig. 2). The test years 2011 (*PA* = 0.06 ± 0.01) and 2018 (*PA* =  −0.04 ± 0.03) were poorly predicted across all scenarios (Fig. 2).

## Discussion

Here, we evaluated linear and semi-parametric methods using genomic, pedigree, and QTL information for genomic prediction of resistance to stripe rust across 11 years in an Austrian winter wheat breeding program. Resistance to stripe rust in an active wheat breeding program is partially influenced by the combination of two dynamic processes: (a) breeders’ decisions about family selection at every generation/year and (b) rapidly changing *Pst* populations. We found small differences in performance among prediction models and that cross-validated predictive ability was moderate within years but poor in most cross-year scenarios.

GRKHS modeling yielded the best overall predictive ability in the cross-validated framework but the difference between GRKHS/GRKHS-A and the other models was small, with insignificant differences with GBLUP modeling (3–4%) and slightly larger differences with the pedigree-based models (4–10%). Previous studies comparing genomic prediction models for stripe rust resistance in wheat found that GRKHS had similar performance to GBLUP (Juliana et al. 2017; Tehseen et al. 2021) and slightly better accuracy than pedigree-based models (Juliana et al. 2017). GRKHS had greater heritability than all other models under both cross-validated and cross-year prediction, with notable differences ranging from 7 to 45% under cross-validation. Previous studies also found that RKHS methods reduce error variance and capture a greater amount of the genetic variance (Gianola et al. 2006; Crossa et al. 2010) and may improve prediction under epistasis (Gianola et al. 2006; Gianola and Van Kaam 2008; Heslot et al. 2012; Juliana et al. 2017).

As expected given the quantitative inheritance of stripe rust resistance in this population (Morales et al. 2021), OLS had poor predictive ability compared to the genomic and pedigree-based kinship models. While genomic prediction is an effective tool for improving quantitative traits (Heffner et al. 2010; Heslot et al. 2015; Poland and Rutkoski 2016; Crossa et al. 2017), approaches that incorporate individual markers, such as OLS and MAS, can be used successfully under less complex genetic architecture and where major QTL are present (Ragimekula et al. 2013; Poland and Rutkoski 2016; Juliana et al. 2017; Chen 2020; Shahinnia et al. 2022).

The QTL used as prediction model covariates here, which had been previously identified in a GWA study in this population, had small effects on stripe rust resistance (Morales et al. 2021). Small-effect QTL are not ideal targets for MAS, but previous studies have found that the inclusion of small- and medium-effect QTL as prediction model covariates can improve predictive ability for stripe rust resistance (Juliana et al. 2017; Shahinnia et al. 2022). The inclusion of QTL covariates in genomic prediction modeling did not significantly increase predictive ability when compared to the respective models without QTL covariates (e.g., GBLUP vs. GBLUP-A) in our dataset. Our results suggest that background quantitative resistance mechanisms were driving the signal for genomic prediction, complementing previous findings of genome-wide selection signatures in this breeding program (Morales et al. 2021). In addition, Morales et al. (2021) found that rapid changes in allele frequencies led to the fixation of QTL detected by GWA in this population. As such, modeling QTL covariates may not be a reliable approach for long-term improvement of genomic prediction for stripe rust resistance in some breeding programs. The utility of QTL in prediction or MAS for resistance to stripe rust largely depends on the plant material. Breeding programs should—and often do—evaluate different strategies for genomic prediction modeling and/or MAS.

In 2011, the Warrior race (genetic group *PstS7*) emerged across Europe and quickly became the dominant *Pst* race thereafter (Hovmøller et al. 2016; Global Rust Reference Center 2021). Predictive ability and heritability were very poor for 2011 under both cross-validated and forward prediction. In addition, including genotypes that were present in both the training and test years in between-year genomic prediction modeling reduced predictive ability from 2011 to 2013. These results suggest that some resistance alleles in the population may have become ineffective with the emergence of the Warrior race. However, resistance to stripe rust in this breeding program appears to have been largely driven by quantitative mechanisms, as demonstrated by (a) our previous findings of quantitative inheritance and genome-wide selection (Morales et al. 2021), (b) the lack of improvement in genomic prediction accuracy by incorporating QTL covariates, and (c) the non-relationship between proximity in time and predictive ability under cross-year genomic prediction frameworks.

Here, predictive ability for stripe rust resistance was higher under cross-validation than in the cross-year prediction frameworks, similar to previous reports where prediction accuracy for other traits was higher within populations than across populations (Thavamanikumar et al. 2015; Haile et al. 2021; Isidro y Sánchez and Akdemir 2021). Compared to a study on genomic prediction for stripe rust in bread wheat landraces from Afghanistan, we found similar levels of cross-validated predictive ability (Tehseen et al. 2021), while our cross-validated genomic predictive ability results were lower than those reported in advanced lines from the CIMMYT bread wheat program (Juliana et al. 2017) and in a panel of Central European winter wheat (Shahinnia et al. 2022). The higher cross-validated predictive ability in previous studies may have been the result of more highly controlled, replicated, and artificially inoculated experiments (Juliana et al. 2017; Shahinnia et al. 2022).

The data used in this study was distinct from previous experiments in that (a) it derived from an active breeding program, in which more than 5000 genotypes were evaluated, with breeders’ decisions leading to rapid genetic changes in the population over time and (b) the trials were conducted over 11 years at more than 50 locations, largely under natural *Pst* infection. In addition, all previous studies on genomic prediction modeling for stripe rust resistance have been conducted within populations, while our work has assessed models under both cross-validated (within-year/population) and cross-population (forward and between-year) frameworks (Juliana et al. 2017; Muleta et al. 2017; Tehseen et al. 2021; Shahinnia et al. 2022). Forward and between-year genomic prediction was poor, while phenotypic correlation between pairs of years was moderate. In addition, including genotypes that were observed in both the training and test years in genomic prediction modeling decreased predictive ability in the cross-year frameworks. Spatiotemporal changes in *Pst* population composition can lead to changes in observed levels of stripe rust resistance, as resistance alleles can break down with genetic changes in the pathogen (Michel et al. 2022). The complex pathosystem between *Pst* and wheat, especially in an active breeding program, makes genomic prediction for stripe rust challenging in the long term.

Our results suggest that although cross-validated, within-environment prediction can appear promising, genomic prediction across years and germplasm, which would be a more realistic application in a breeding program, may not be sufficient for selection of resistance to stripe rust alone. Screening germplasm for stripe rust resistance in multi-environmental trials is crucial for making informed selection decisions. Although visual phenotypic assessment of stripe rust resistance is less expensive than genotyping, conducting trials across multiple locations/years can be costly (e.g., labor, field space, seed availability) and environmental conditions are not always conducive for *Pst* infection and stripe rust symptom development, even under artificial inoculation. As such, selective phenotyping and genotyping strategies should be optimized within breeding programs to maximize the efficiency of selection for stripe rust resistance.

### Supplementary information

**Online Resource 1.** Genotype best linear unbiased estimates (BLUEs) for stripe rust resistance within and across years from 2008 to 2018.

**Online Resource 2.** Pedigree information for 41,461 lines, including genotype IDs of each line and of each parent.

**Online Resource 3.** Data for 9744 SNPs, including SNP ID, chromosome, physical position (bp), and genotypes of 5233 lines.

**Online Resource 4.** Information for 64 QTL used as covariates in prediction models, including SNP ID, chromosome, physical position (bp), and year in which the QTL was identified.

## Supplementary Information

Below is the link to the electronic supplementary material.

Supplementary file1 (TXT 848 KB)

Supplementary file2 (TXT 1006 KB)

Supplementary file3 (TXT 116197 KB)

Supplementary file4 (TXT 2 KB)

## Data Availability

All phenotypic, genotypic, and pedigree data and results from the analyses presented here are included in the manuscript materials.

## Code availability

The scripts used to conduct the analyses presented here are available upon request.
